# Effects of a Mobile Storytelling App (Huiyou) on Social Participation Among People With Mild Cognitive Impairment: Pilot Randomized Controlled Trial

**DOI:** 10.2196/70177

**Published:** 2025-06-18

**Authors:** Di Zhu, Abdullah Al Mahmud, Wei Liu

**Affiliations:** 1 Centre for Design Innovation Swinburne University of Technology Melbourne Australia; 2 School of Design Southern University of Science and Technology Shenzhen China

**Keywords:** digital storytelling, technology-based intervention, social participation, people with mild cognitive impairment, digital health, mobile apps

## Abstract

**Background:**

As the prevalence of mild cognitive impairment (MCI) among older adults increases, so does the need to enhance social participation and cognitive functions through innovative interventions. Digital storytelling in group settings holds potential not only to foster social connections but also to integrate with traditional in-person activities, leveraging both for greater impact.

**Objective:**

This study aimed to evaluate the feasibility of the Huiyou app in supporting group-based storytelling activities, aiming to enhance social participation for people with MCI. We focused on the app’s ability to improve storytelling goal attainment, social connectedness, self-efficacy, and subjective happiness, comparing these outcomes between the experimental and control groups.

**Methods:**

We randomly assigned 20 participants with MCI to either an intervention group or a control group, engaging them in the use of the Huiyou digital storytelling app over 4 weekly sessions of 45 minutes each. We measured outcomes through the Assessment of Life Habits questionnaire (77 items), particularly outdoor activities and interpersonal relationships; the Social Connectedness Scale–Revised (20 items); the General Self-Efficacy Scale (10 items), focusing on coping self-efficacy; and the Subjective Well-Being Scale (SWBS; 20 items), with a special emphasis on self-acceptance.

**Results:**

The sample had an average age of 69.7 (SD 3.21) years, with no significant (*P*=.23) baseline differences between groups in age, sex, or educational background. Cognitive function, assessed via the Montreal Cognitive Assessment–Chinese questionnaire, also showed no significant differences at baseline (*P*=.20). Specifically, significant enhancements in the outdoor activity (mean value difference 0.171, SD 0.353; Cohen *d*=1.046; *P*=.03) and interpersonal adaptation experience subscales of the SWBS (mean value difference 0.167, SD 0.247; Cohen *d*=1.290; *P*=.01) were noted. Notably, storytelling performance improved markedly, evidenced by increases in story sharing duration and complexity. Although overall improvements in Assessment of Life Habits (*P*=.14), Social Connectedness Scale–Revised (*P=*.59), and Subjective Well-Being Scale (*P*=.26) scores were not statistically significant, the large effect sizes observed suggest potential benefits of the Huiyou app that might be obscured by the study’s small sample size.

**Conclusions:**

This study indicates that the Huiyou mobile storytelling app is feasible to enhance social participation and specific aspects of social functioning such as interpersonal adaptation for people with MCI. Despite the lack of significant changes in overall scores for key scales, observed effect sizes highlight a positive trend that merits further investigation. These results advocate for the continuation of digital intervention development to improve quality of life and social integration for individuals with MCI.

## Introduction

### Background

People with mild cognitive impairment (MCI) face significant challenges in daily life due to cognitive deficits and emotional changes that reduce their ability to function socially [1]. Cognitive decline is closely linked to decreased social activities, with behavioral symptoms and social-cognitive deficits often leading to poor maintenance of social networks and difficulties using everyday technology [2,3]. Memory loss further undermines feelings of security and autonomy, often resulting in frustration and anxiety [4]. Despite these challenges, maintaining high-level social participation is crucial for people with MCI as it has been shown to prevent or slow down cognitive decline, improve quality of life, and reduce social isolation and loneliness [5-8]. Despite this, people with MCI often experience reduced social connections and activities, which are fundamental to social participation [9,10]. Conversely, limited social engagement can lead to further cognitive decline and increased isolation [11,12]. However, multiple barriers, including the subtle nature of MCI-related social decline, often impede participation in social activities. In response, various interventions have been developed to support social participation for people with MCI. Technology-based interventions, supported by modern technologies such as cameras, internet communication, and speech-to-text tools, have shown promise in enhancing social participation for people with MCI. These interventions include cognitive rehabilitation programs, educational tools, telecommunication systems, social robots, and self-management systems, all designed to improve social engagement and quality of life [13-15]. While some studies suggest that these interventions have outcomes comparable to those of traditional treatments, such as in the quality of social interactions [16], they offer the advantage of expanding care from hospitals to homes, reducing therapists’ workload and enabling remote treatment [17]. Recent studies have demonstrated that patients engaging in digital technology–based serious game interventions experience notable improvements across multiple domains, including global cognitive function, executive function, attention, mood (notably reductions in depression), and activities of daily living, compared to those undergoing traditional rehabilitation or receiving standard medical care [18]. Given this context, digital storytelling interventions, which combine the benefits of technology with the therapeutic power of narrative, hold particular potential to further enhance social participation and cognitive engagement among individuals with MCI.

Digital storytelling is a powerful tool for enhancing cognitive engagement and fostering social connections, particularly among older adults with MCI. Engaging in storytelling requires individuals to recall past experiences, organize their thoughts, and articulate them coherently [19], which stimulates essential cognitive processes such as memory recall, attention, and language skills. For those with MCI, this activity offers a structured yet creative outlet for mental engagement, helping maintain and even improve cognitive functions that are often challenged by the condition [20]. In addition, storytelling fosters social connections by creating opportunities for individuals to share their personal narratives with others whether in person or through digital platforms [21]. Recent developments such as time travel tours have made digital storytelling more accessible to older adults by enabling them to create and share location-based narratives through a conversational web interface without requiring advanced technical skills [22]. This exchange of stories not only builds empathy, understanding, and a sense of community but also combats the social isolation that frequently accompanies cognitive decline [23]. When older adults share their stories in a group setting, they reinforce their own memories while contributing to the collective memory of the group, further strengthening social bonds [24]. As an intervention, digital storytelling uniquely addresses the cognitive and social challenges faced by people with MCI by combining the therapeutic benefits of storytelling with the accessibility and engagement potential of digital tools. This dual focus on cognitive engagement and social connection makes digital storytelling a valuable approach for improving the overall well-being of older adults with MCI, providing them with a meaningful way to connect with others and exercise their cognitive abilities.

### Prior Work

Technology-based storytelling interventions involve recalling previous actions, events, and feelings using physical prompts (ie, photographs, household items, music, and audio recordings). Such prompts are now commonly stored and presented digitally [25]. However, there is some evidence, albeit not conclusive, suggesting that storytelling interventions or reminiscence therapy for dementia yield no significant effect on quality of life immediately following the intervention period when compared to a control group (no treatment; see the study by Wood [26]). In reviews of storytelling interventions and apps aimed at enhancing social participation among people with MCI [19,27], we identified 4 key themes: capture memory, saving memory, memory retrieval, and sharing. These themes represent the core aspects of the interventions, focusing on how individuals can record, store, recall, and share their personal experiences. These elements collectively contribute to the effectiveness of technology-based storytelling interventions.

Various digital storytelling applications are designed to assist older adults, particularly those with MCI or dementia, in enhancing their mood and increasing social engagement [28]. The main features for generating materials across these applications include the ability to upload personalized content such as photos, videos, and music [29]. Some applications offer additional capabilities such as selecting auto-play intervals [30], adding tags [31], or creating dynamic timelines [29]. For memory retrieval, features include auto-playing photos and videos, viewing materials on timelines, and using virtual reality to project realistic images [32]. Some applications support a random display of materials to trigger reminiscence [33]. Story sharing capabilities vary, with some applications enabling sharing through smartphones, tablets, or dedicated platforms [34,35]. These features often include options to share edited storybooks, personal videos, or narrated slideshows with family, friends, or broader networks [25,30]. Additional features frequently involve creating structured stories, using prewritten scripts, integrating multimedia content, and supporting administrative activities such as tagging and organizing materials [36].

The platforms for these applications are primarily smartphones and tablets [32], with some using laptops, computers, or virtual reality devices [37]. The impact on social participation includes improving mood, increasing social interactions, enhancing subjective well-being, and fostering closer ties with friends and family [30]. However, specific outcomes vary, and not all applications provide detailed results on social participation. Overall, these digital storytelling applications demonstrate significant potential for enhancing social engagement and emotional well-being among older adults with cognitive impairments.

Digital storytelling interventions offer a promising avenue for enhancing social participation among people with MCI, but they also face significant barriers and limitations. One major issue is that most interventions target individual relationships rather than broader community-based connections [8,38], which can limit the social impact and scope of interaction. This focus on personal relationships might support social connections within families but does not necessarily encourage engagement with the wider community or strangers, which is essential for comprehensive social participation. Another barrier involves the difficulty that people with MCI often encounter with commercial digital storytelling applications [39]. These applications frequently require a level of digital literacy that can be challenging for this group even after training sessions. As a result, many interventions rely on caregivers or trained volunteers to help develop and share stories, which may not be sustainable or widely accessible [29,40]. This indicates a need for user-friendly applications designed to accommodate the specific needs and capabilities of people with MCI, reducing the learning curve and associated resource demands. There is a research gap in developing customized software that caters specifically to the usability needs of people with MCI [41,42]. The addition of group elements to digital storytelling can enhance engagement and foster a sense of community [43], yet current digital storytelling tools focus on individual experiences, not collaborative storytelling [30,44]. Privacy concerns also emerge when sharing stories with broader audiences [43,45]. While sharing personal stories online can create new connections and engagement, it also poses risks of exposing sensitive information or triggering unfavorable reactions from a wider audience. This necessitates careful consideration of privacy and safeguarding measures when designing digital storytelling interventions. Moreover, most studies use the basic versions of the interventions or applications, but few studies have been conducted as randomized controlled trials to measure the efficacy of the interventions or applications [19]. Recent international research underscores the potential of digital health interventions in supporting individuals with MCI. A systematic review and meta-analysis by Di Lorito et al [46] found that such interventions can produce positive effects on cognitive abilities among people with MCI and dementia. Furthermore, Karakose et al [47] conducted a comprehensive bibliometric and science mapping analysis, revealing the evolving landscape of digital addiction research, which is pertinent to understanding user engagement with digital health tools. These findings highlight the importance of integrating international perspectives to inform the development and implementation of digital storytelling applications such as Huiyou.

### The Huiyou App

To bridge these research gaps, we co-designed an app, 会友 (Huiyou, derived from the pinyin pronunciation, which means “meeting new friends” in Chinese), with therapists and people with MCI [48]. It is inspired by a classic quote from Confucius in the *Analects*: “A gentleman seeks friendship through literature and reinforces goodness through friendship.” This statement emphasizes the idea that individuals cultivate friendships through literary exchange and support virtue through companionship. The name reflects a positive vision of fostering social connections through literature, friendship, and benevolence. Users can leverage technological means through the software to expand their social circles, facilitating deeper communication and connections with others. The name embodies the social nature of the software and its goal of promoting friendship. Huiyou effectively stores memories from daily life and encourages people with MCI to reminisce and discuss favorable memories with new friends. Huiyou has 2 main features: supporting people with MCI to conduct self-reflection daily (preparing materials using cues) on certain topics and facilitating group memory retrieval (presenting a story and promoting a discussion with group members). In addition, Huiyou uniquely integrates structured outdoor activities with digital storytelling to reinforce physical engagement alongside cognitive and social interaction. The 2 innovative aspects of Huiyou are embedded memory retrieval for capturing daily life and its ability to collect recent, valuable memories. The intervention design also deliberately combines self-generated memory prompts with shared group discussions to maximize both personal reflection and collective participation. Another feature is combined self-reflection and group reflection to enhance social interaction during the discussion in addition to sharing and participating in social activities outside the home. A previous publication provides more details about the design of Huiyou [48].

### Study Objectives

This study contributes theoretically by advancing the understanding of digital storytelling to enhance social participation and emotional well-being for people with MCI. Empirically, it delivers one of the first pilot evaluations of a co-designed, culturally adapted mobile storytelling app for community-dwelling older adults with MCI in China. The integration of storytelling with structured outdoor engagement provides a practical model for accessible, technology-supported interventions in low-resource settings. This experimental study aimed to investigate the feasibility of the Huiyou digital storytelling intervention for people with MCI. We hypothesized that (1) the Huiyou intervention would significantly enhance social participation among individuals with MCI, as evidenced by improved quantitative measures of social participation outcomes; and (2) the intervention would facilitate the achievement of predefined social participation goals, with quantitative assessments confirming goal attainment.

## Methods

### Ethical Considerations

#### Ethics Review Approvals or Exemptions

Ethics approval for this study was granted by the Swinburne University of Technology Human Research Ethics Committee (approval number 20226525-11105; September 26, 2022). The study was conducted in accordance with the principles of the Declaration of Helsinki. The study protocol was also published in advance [49].

#### Informed Consent

Informed consent was obtained from all participants prior to their involvement in the study. For participants with mild cognitive impairment (MCI), consent was obtained with the support of a formal or informal caregiver. Participants were also asked to articulate their reasons for participation to confirm understanding and willingness. Trained research assistants provided step-by-step guidance throughout each session, addressing confusion or distress in real time. Participants were continuously monitored for signs of cognitive overload or discomfort, with immediate support provided as needed.

#### Privacy and Confidentiality

All data were anonymized using participant ID codes, and no personally identifiable information was collected or stored. Data were securely stored on encrypted servers with access restricted to authorized research personnel. No identifiable images or personal data of participants are presented in this manuscript.

#### Compensation Details

No financial or material compensation was provided to participants. Participation was entirely voluntary, and support was offered to ensure a safe and respectful experience.

### Study Design and Procedure

We conducted a 4-week experimental study with a waitlist control group to assess the impact of the Huiyou app on people with MCI (see Multimedia Appendix 1 for the CONSORT checlist). The randomization sequence for participant allocation was computer generated and managed through a Microsoft Excel spreadsheet. As this study involved active participation in the experimental activities, it was designed as a single-blind experiment. The experiment was conducted in 2 batches, with the second batch serving as a supplemental recruitment due to some participants’ limited availability.

This study commenced with the installation and learning of the Huiyou software, along with pretesting, on December 18, 2023. The intervention period spanned the second to the fifth week, with posttesting conducted at the end of the fifth week and some participants opting for testing in the sixth week. The intervention sessions took place at the Jing Shi Psychology Centre Meeting Room in Tiantongyuan, Tongzhou District, Beijing.

After each intervention session, the theme for the following week was announced, with the first week’s theme disclosed after the pretest. A WeChat group was established to facilitate the convenient communication of activity information and scheduling adjustments. Participants had the flexibility to upload the necessary materials for their stories through the Huiyou app at any time, including photos and videos.

### Recruitment

**Figure 1:**
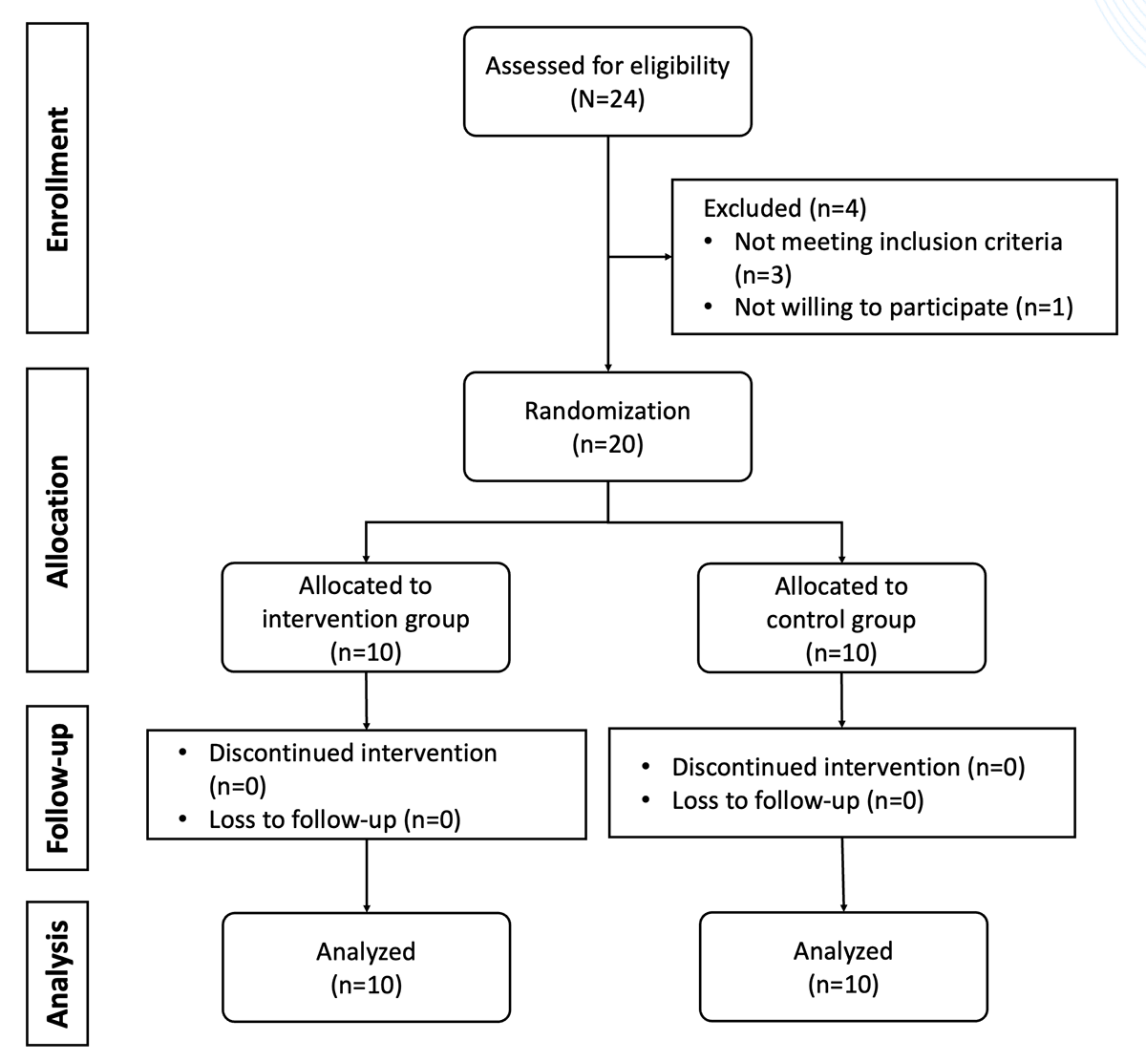
Flowchart for selecting the study participants.

Recruitment for this study commenced in September 2023 and concluded in December 2023. We initiated participant selection by prescreening people with MCI from previous studies. Our collaboration with the Jing Shi Psychology Centre was pivotal in reaching out to potential participants. The center provided these individuals with detailed information about the study, including providing informed consent forms and an overview of the activities involved. A total of 20 participants enrolled in the study, with 10 (50%) randomized to the experimental group and 10 (50%) to the control group (Figure 1). The communication between the participants and staff in the partner institution before the formal research was aimed at gauging their interest in participating in our research. In addition, the center assisted in the recruitment process by conducting screenings of people with MCI using the Montreal Cognitive Assessment–Chinese (MoCA-C) questionnaire [50]. This meticulous and collaborative approach ensured that we recruited participants who met the study criteria and were well informed and motivated enough to contribute to our research on improving the lives of people with MCI. Participants were asked to complete the scales within a week.

The inclusion criteria for recruiting people with MCI were being clinically diagnosed with MCI using the MoCA-C screening tool, residing in community-dwelling complexes in Beijing, being aged ≥65 years, and having no significant visual or hearing impairments. In addition, participants were required to have adequate reading skills, especially for digital interfaces. Exclusion criteria were in place for individuals who had neurological conditions such as stroke or brain injury as these could affect daily functioning and impact study outcomes.

### Intervention: Huiyou Group

Huiyou is an Android-based mobile app designed to support digital storytelling co-designed with therapists and people with MCI [48]. Participants engage with weekly activity themes, where they are prompted to prepare and upload photos or videos relevant to 1 of 3 selected themes. Users can also edit keywords and categorize them for better organization. Once an activity begins, participants are grouped into small teams of 3 to 4 members for a storytelling session. A tablet serves as the interface for the storyteller, displaying prepared keywords to aid in their narrative, whereas a computer screen acts as the display terminal for the audience. Currently, owing to technical constraints, the display technology is managed through Keynote (Apple Inc), with researchers predownloading and processing materials to ensure smooth playback. Participants are given approximately 30 minutes before each activity to prepare their materials. The sessions are held weekly, with the timing determined by each group through discussions before each activity. Goal assessments are conducted at the end of the second and fourth weeks, with detailed records kept of goal completion, notes, and the duration of each story shared.

**Figure 2:**
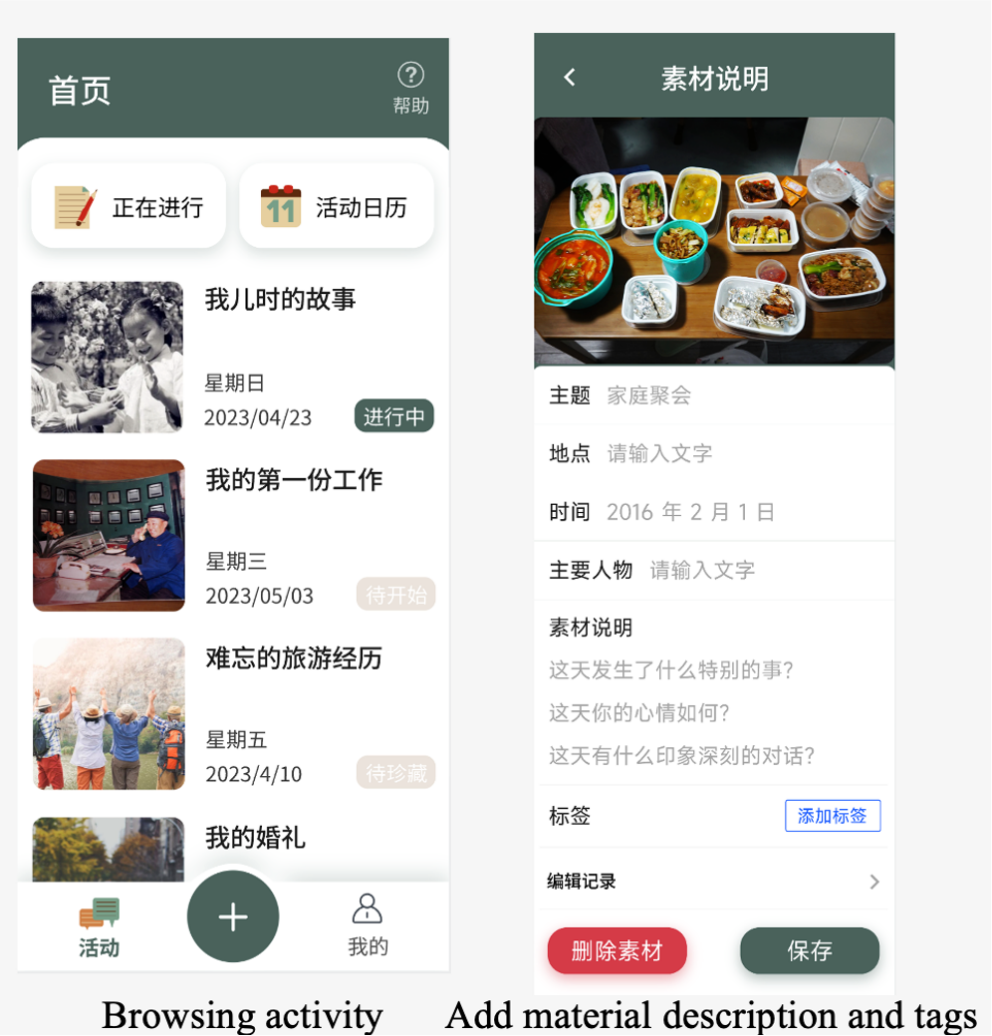
Screenshots of Huiyou—memory collection.

**Figure 3:**
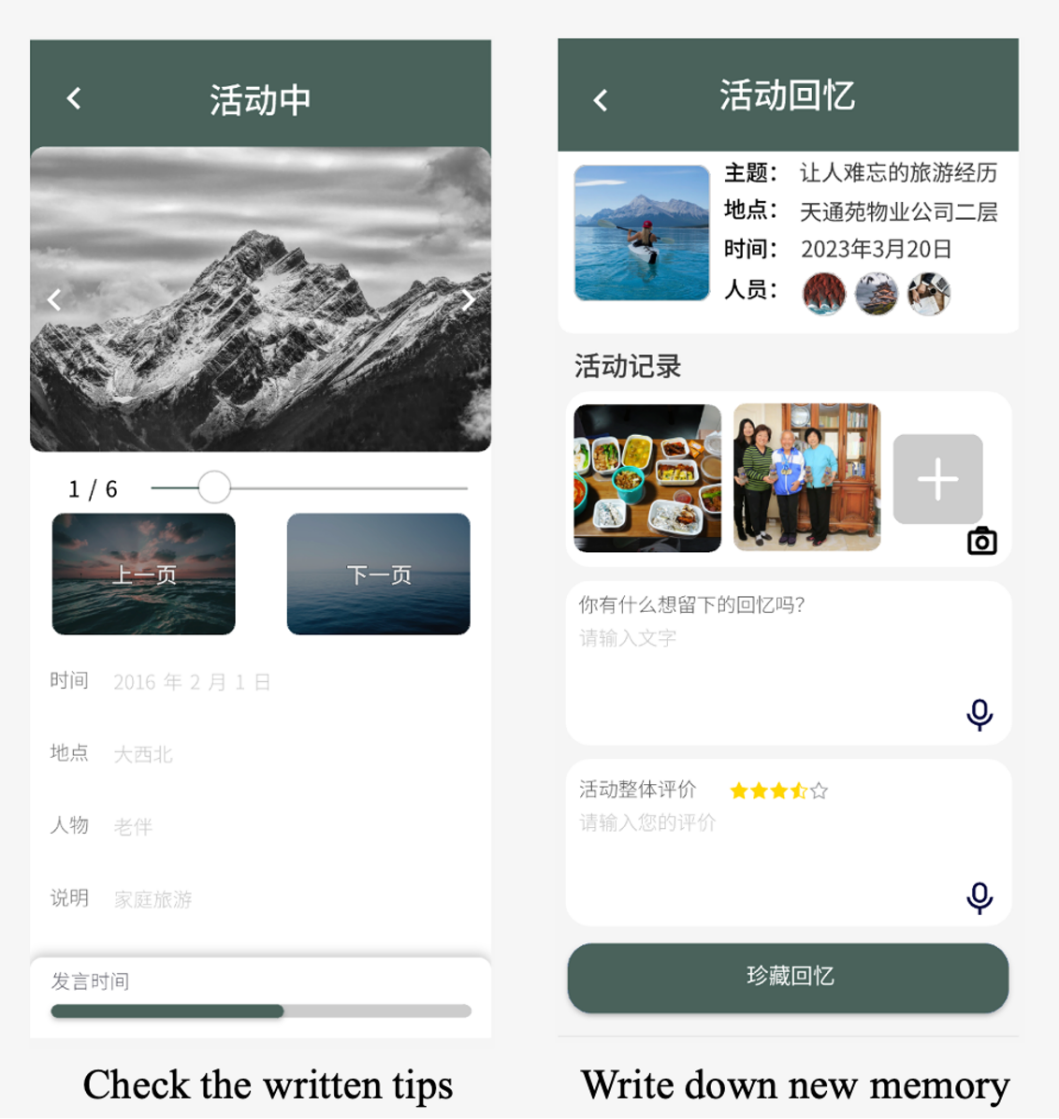
Screenshots of Huiyou—story sharing.

Figures 2 and 3 illustrate various functionalities of the Huiyou mobile app designed to enhance user interaction and memory recording. The workflow of Huiyou is as follows. Participants sign up for topics of interest, and we assist them in scheduling a sharing time. Huiyou assigns 3 missions, providing several guiding questions to help participants think about and prepare their materials in advance. During preparation, participants can type in memos and tips. During the sharing session, the pictures are displayed to group members, and participants can refer to their memos. This process ultimately creates a shared storytelling memory. The co-design and usability study of Huiyou provides a more detailed description of the app [48]. The first screenshot in Figure 2 shows the Browsing Activity page, offering a user-friendly interface for users to navigate through different activities seamlessly. The second screenshot in Figure 2 highlights the functionality for users to add material descriptions and tags, enabling them to organize and categorize their entries for better accessibility and management. The first image in Figure 3 shows the Check the Written Tips section, where users can access practical advice on using the app effectively. Finally, the Write Down New Memory feature, which provides a structured format for users to document new memories with prompts and text fields to guide detailed recollections, is shown in the second screenshot of Figure 3.

### Waitlist Control Group

The participants in the waitlist control group were involved in all evaluations at the beginning (T0) and end (T1) of the study. After completing the final evaluation (T1), they were offered the opportunity to learn about the Huiyou program, which they could potentially use in future research. This arrangement ensured that all participants received equal information about the program’s features and potential benefits, although they did not use the program during the study period [49].

### Measures

Assessments were completed at community locations in Beijing, China, at baseline (T0) and 4 weeks (T1). Baseline participant characteristics included age, sex, educational level, and MoCA-C score. This paper summarizes the assessments for social participation (Assessment of Life Habits [LIFE-H]; 77 items), social connectedness (Social Connectedness Scale–Revised [SCS-R]; 20 items), self-efficacy (General Self-Efficacy Scale [GSES]; 10 items), and subjective sense of happiness (Subjective Well-Being Scale [SWBS]; 20 items), as Table 1 shows. Further details of the study design can be found in the protocol paper [49].

**Table 1:** Study assessments and outcome measures, which were conducted on all participants at baseline and after 4 weeks.

Assessment	Outcome
Social participation	The LIFE-H^a^ (77 items): indoor activity, outdoor activity, interpersonal relationships, community life, and amusement and recreation
Goal attainment of storytelling	Duration of story sharing Number of key events in the story Number of photos collected Number of notes made on the photos
Social connectedness	SCS-R^b^ (20 items)
Self-efficacy	GSES^c^ (10 items): action self-efficacy and coping self-efficacy
Subjective sense of happiness	SWBS^d^ (20 items): interpersonal adaptation experience, mental health experience, family atmosphere experience, psychological balance experience, physical health experience, target value experience, social confidence experience, growth and progress experience, contented and abundant experience, and self-acceptance experience

^a^LIFE-H: Assessment of Life Habits.

^b^SCS-R: Social Connectedness Scale–Revised.

^c^GSES: General Self-Efficacy Scale.

^d^SWBS: Subjective Well-Being Scale.

All the scales used have validated Chinese versions, and we used these versions in this study. Social participation was evaluated using the Chinese version of the LIFE-H, a validated and reliable self-report questionnaire comprising 77 items [51]. This instrument assesses various aspects of daily living, including indoor activities, outdoor activities, interpersonal relationships, community life, and amusement and recreation. Goal attainment in storytelling was measured by recording several key metrics: the duration of story sharing, the number of key events included in each story, the number of photos collected, and the number of annotations made on these photos. Participants set baseline targets on a 10-point scale before commencing the activity and revisited these targets during the second and fourth weeks of the intervention to track progress and achievement [52]. Social connectedness was assessed using the SCS-R, a 20-item, well-established self-report questionnaire known for its validity and reliability in measuring individuals’ sense of belonging and connection to others [53]. Self-efficacy levels were measured using the GSES, a validated 10-item questionnaire that evaluates both action self-efficacy and coping self-efficacy, reflecting the participants’ confidence in managing and executing tasks as well as handling challenging situations effectively [54,55]. The subjective sense of happiness was evaluated using the SWBS, another reliable and valid self-report instrument consisting of 20 items. This scale captures a wide range of experiences related to well-being, including interpersonal adaptation, mental health, family atmosphere, psychological balance, physical health, goal achievement, social confidence, personal growth, contentment, and self-acceptance [56]. Interpersonal adaptation is the process of adjusting one’s behavior and communication style to better fit in with others in social interaction [53].

In addition to the structured quantitative assessments, qualitative observations were conducted during the field-testing sessions to capture participants’ engagement and social interaction patterns. Researchers took brief field notes after each session, documenting participants’ verbal and nonverbal responses, interactions, and levels of engagement. These observations were intended to supplement the quantitative findings by offering contextual insights into the participants’ experiences with the intervention [57]. A full systematic analysis of these qualitative data was outside the scope of this paper and has been reported separately in a companion article focusing specifically on the qualitative findings. In this paper, qualitative observations are referred to selectively to support the interpretation of key quantitative outcomes.

### Statistical Analysis

Quantitative data analysis was conducted in the JASP software [58]. The analysis focused on key metrics related to the digital storytelling intervention: duration of story sharing, number of key events in the story, number of photos collected, and number of notes made on the photos. Participants submitted subjective reports at 3 junctures: baseline, the 2-week follow-up, and the 4-week follow-up. For each metric, we followed the Bangor Goal-Setting Interview Manual [52] to calculate the mean and variance of and changes in the outcomes reported by participants in addition to objective measures such as the actual duration of story sharing, the total photos collected, and the tally of notes made. To standardize the scoring across various metrics, a normalization method was applied: the maximum number of photos (n=9) was set as a perfect score of 10, with each participant’s count proportionately scaled. Similarly, for the duration of story sharing and the number of notes, the highest recorded value was established as the benchmark for a full score of 10, with other values adjusted accordingly. Regarding the number of key events in the story, following transcription, our research team conducted a quantitative assessment to enumerate significant narrative elements within each shared story. Finally, to attain a comprehensive insight into the participants’ experiences and the intervention’s impact, thematic coding was applied to analyze the data from the concluding user interviews.

To evaluate the intervention’s impact, we calculated a standardized change rate for each outcome measure:



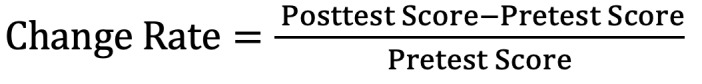



This calculation standardizes each participant’s change relative to their baseline, providing a more sensitive measure of group differences. Independent *t* tests were conducted in JASP to compare these change rates between the intervention and control groups, with *P* values and effect sizes with 95% CIs reported. An α level of .05 was set for all 2-tailed tests. No interim analyses or stopping rules were applied, as the trial involved a small sample and posed minimal risk to participants. Participants and facilitators were not blinded due to the nature of the intervention. However, outcome assessors and data analysts were blinded by using coded group labels and de-identified data.

All randomized participants (N=20) were included in the final analysis and analyzed in the groups to which they were originally assigned, following an intention-to-treat approach. No participants were lost to follow-up. Minor missing data identified during data checking were subsequently completed through verification with source records, and no imputation was required. No subgroup or sensitivity analyses were prespecified or conducted, given the exploratory nature of the study. Harms were described narratively, as no adverse events were reported.

### Harms Assessment

Given the non-invasive and low-risk nature of the digital storytelling intervention, no formal adverse event monitoring was implemented. Harms were defined broadly as any negative physical, emotional, or psychological responses associated with participation in the intervention. These were assessed non-systematically through participant self-reports and informal observations made by facilitators during and after each session. No adverse events or participant-reported harms were observed or reported throughout the trial.

## Results

### Sample Characteristics

A total of 20 participants were recruited and randomly assigned to either the intervention group or the waitlist control group (n=10, 50% per group). Demographic and cognitive assessments showed no significant differences between the groups, supporting the randomization protocol (Table 2). Participants were randomly assigned to the intervention or control group in a 1:1 ratio using simple randomization. The allocation sequence was generated by an independent researcher using Excel’s RAND function and implemented via sequentially numbered, opaque, sealed envelopes. The personnel responsible for enrolling participants and assigning interventions did not have access to the allocation sequence, ensuring allocation concealment. Specifically, the difference in age was not statistically significant (*P*=.23), nor were differences in sex distribution (*P*=.29), educational attainment (*P*=.41), or MoCA-Ca scores (*P*=.20). Participants averaged 69.7 (SD 3.21) years of age, with no significant difference between groups (mean 68.80, SD 2.61 in the intervention group vs mean 70.60, SD 3.49 in the waitlist control group; *P*=.23). Most participants (16/20, 80%) were female, with sex distribution consistent across groups (*P*=.29). Educational levels and cognitive function (assessed using the MoCA-C) did not significantly differ between groups (*P*>.05 in all cases).

**Table 2:** Sample characteristics overall and by treatment condition (N=20).

Characteristic		Total	Intervention (n=10)	Waitlist control (n=10)	*P* value	
Age (y), mean (SD; range)		69.7 (3.21; 65-76)	68.80 (2.61; 65-76)	70.60 (3.49; 65-74)	.23	
**Sex, n (%)**						.29
	Male	4 (20)	1 (10)	3 (30)		
	Female	16 (80)	9 (90)	7 (70)		
**Educational level, n (%)**						.41
	University	4 (20)	2 (20)	2 (20)		
	High school	7 (35)	5 (50)	2 (20)		
	Middle school	9 (45)	3 (30)	6 (60)		
MoCA-C^a^ score, mean (SD)		23.25 (1.51)	23.7 (1.19)	22.8 (1.66)	.20	

^a^MoCA-C: Montreal Cognitive Assessment–Chinese version.

### Intervention Impact Analysis

**Figure 4:**
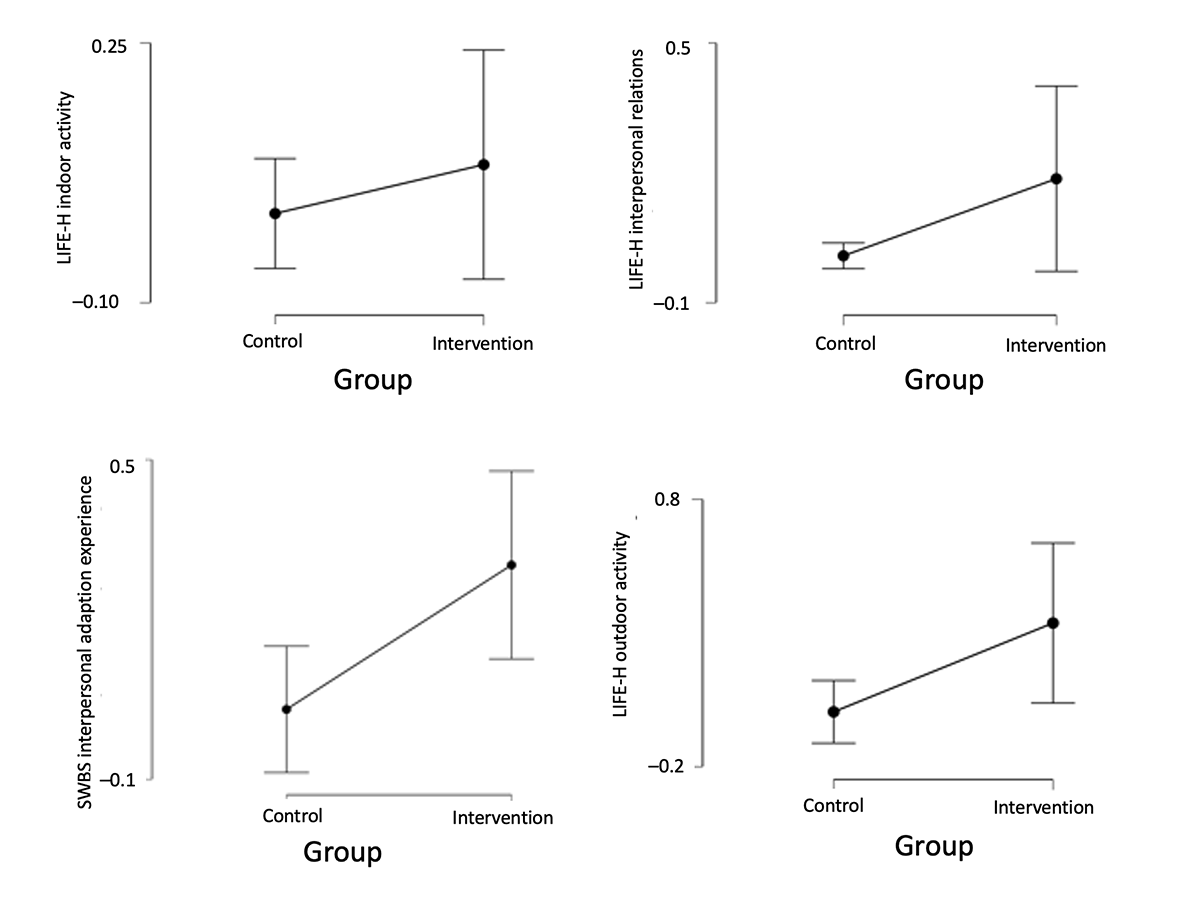
Selected outcome measure change rates—intervention versus control group. LIFE-H: Assessment of Life Habits; SWBS: Subjective Well-Being Scale.

According to statistical comparisons of change rates across key outcome measures (Multimedia Appendix 2), significant improvements were observed in outdoor activity on the LIFE-H (t_18_=2.339; Cohen *d*=1.046; *P*=.03) and in interpersonal adaptation experience on the SWBS (t_18_=2.884; Cohen *d*=1.290; *P*=.01), indicating that the intervention effectively enhanced participants’ engagement in outdoor activities and interpersonal adaptation. In other measures, such as indoor activity on the LIFE-H (*P*=.40) and the GSES (*P*=.98), slight but nonsignificant improvements were noted. Figure 4 illustrates the change rates across outcome measures, with clear improvements for outdoor activity and interpersonal adaptation, whereas other measures showed overlapping trends, consistent with the nonsignificant *t* test results.

### Effect of Huiyou

To assess specific domains of social participation and subjective well-being, we used the LIFE-H, SCS-R, GSES, and SWBS. Pre- and postintervention *t* tests were conducted to compare group differences (Multimedia Appendix 3). Most measures showed no significant baseline differences between the experimental and control groups, confirming initial equivalence. Specifically, baseline comparisons showed no significant group differences in general LIFE-H (*P*=.14), indoor activity (*P*=.40), outdoor activity (*P*=.03), interpersonal relationships (*P*=.08), community life (*P*=.81), SCS-R (*P*=.59), GSES total (*P=*.98), action self-efficacy (*P*=.39), coping self-efficacy (*P*=.14), SWBS total (*P*=.26), interpersonal adaptation (*P*=.01), mental health (*P*=.42), family atmosphere (*P=*.41), psychological balance (*P*=.22), physical health (*P*=.21), target value (*P*=.21), social confidence (*P*=.46), and contented and abundant experience (*P*=.70). However, a significant baseline difference was observed in the self-acceptance experience on the SWBS, where a baseline difference (*P*=.007) was noted and considered in subsequent analyses.

Table 3 presents descriptive statistics for improvement rates across study measures. For LIFE-H categories, average improvement rates ranged from 0.045 (SD 0.156; general) to 0.171 (SD 0.353; outdoor activities), reflecting varied enhancements in daily living habits. The SCS-R showed a mean improvement of 0.098 (SD 0.192). Mental health experience on the SWBS demonstrated the largest variability (mean 0.448, SD 1.12; range −0.222 to 5), indicating individual differences in postintervention outcomes. Other subjective well-being domains, including interpersonal adaptation (mean 0.167, SD 0.247; *P*=.01) and psychological balance (mean 0.313, SD 0.514; *P*=.22), also showed positive trends; however, these results were not statistically significant.

Multimedia Appendix 2 shows the effect sizes (Cohen *d*) of improvement rates across various outcome measures, visually highlighting the magnitude of changes observed between the intervention and control groups. The red dashed line at 0.80 indicates the threshold for a large effect. As shown, interpersonal adaptation experience and outdoor activity demonstrated large effect sizes (Cohen *d*=1.29 and 1.05, respectively), suggesting substantial improvements attributable to the intervention. Several other outcomes, including overall LIFE-H, SCS-R, and SWBS scores, showed medium to small effects, whereas a few measures exhibited minimal or negative effects. This visual summary supports the interpretation that, while the intervention had varied impacts, it significantly enhanced certain aspects of social participation and well-being in people with MCI.

The remaining variables, including different facets of life habits (LIFE-H), general self-efficacy (GSES), social connectedness (SCS-R), and various dimensions of subjective well-being (SWBS), did not show statistically significant changes. Small to moderate effect sizes were observed, suggesting that, while certain domains such as outdoor activity and interpersonal adaptation showed meaningful improvements, other areas such as community life, self-efficacy, and overall subjective well-being exhibited minimal to no change.

**Table 3:** Descriptive statistics and improvement rates in study measures.

			Improvement rate, mean (SD; range)		*t* test (*df*)		*P* value		Cohen *d*
**LIFE-H^a^ general**			0.045 (0.156; −0.273 to 0.597)		1.566 (18)		.14		0.701
	Indoor activity	0.053 (0.168; −0.181 to 0.653)		0.87 (18)		.40		0.389	
	Outdoor activity	0.171 (0.353; −0.2 to 1.414)		2.339 (18)		.03		1.046	
	Interpersonal relationships	0.097 (0.227; −0.02 to 0.923)		1.864 (18)		.08		0.834	
	Community life	0.06 (0.444; −0.56 to 1.778)		0.242 (18)		.81		0.108	
SCS-R^b^			0.098 (0.192; −0.174 to 0.533)		−0.548 (18)		.59		−0.245
**GSES^c^**			−0.006 (0.174; −0.275 to 0.417)		−0.027 (18)		.98		−0.012
	Action self-efficacy	0.012 (0.188; −0.278 to 0.357)		−0.882 (18)		.39		−0.394	
	Coping self-efficacy	−0.015 (0.227; −0.4 to 0.5)		1.562 (18)		.14		0.698	
**SWBS^d^**			0.109 (0.189; −0.103 to 0.603)		1.16 (18)		.26		0.519
	Interpersonal adaptation experience	0.167 (0.247; −0.125 to 0.667)		2.884 (18)		.01		1.29	
	Mental health experience	0.448 (1.12; −0.222 to 5)		0.83 (18)		.42		0.371	
	Family atmosphere experience	0.05 (0.185; −0.125 to 0.714)		0.845 (18)		.41		0.378	
	Psychological balance experience	0.313 (0.514; −0.2 to 2)		1.259 (18)		.22		0.563	
	Physical health experience	0.444 (0.94; −0.2 to 4)		−1.307 (18)		.21		−0.585	
	Target value experience	0.096 (0.247; −0.125 to 0.714)		1.317 (18)		.21		0.589	
	Social confidence experience	0.059 (0.256; −0.4 to 1)		0.762 (18)		.46		0.341	
	Contented and abundant experience	0.01 (0.238; −0.583 to 0.571)		−0.391 (18)		.70		−0.175	
	Self-acceptance experience	0.1 (0.247; −0.2 to 0.714)		−0.283 (18)		.78		−0.127	

^a^LIFE-H: Assessment of Life Habits.

^b^SCS-R: Social Connectedness Scale–Revised.

^c^GSES: General Self-Efficacy Scale.

^d^SWBS: Subjective Well-Being Scale.

### Goal Attainment of Digital Storytelling

Table 4 presents the goal attainment ratings at baseline and the 2- and 4-week follow-ups, alongside statistical comparisons across various measures related to digital storytelling activities among participants. The measures include the duration of story sharing, the number of key events in the story, the number of photos collected, and the number of notes made on the photos, each assessed through participant and objective ratings. At baseline, the duration of story sharing was rated at an average of 4.8 (SD 1.23), which increased to 9.2 (SD 0.79) at the 4-week follow-up. Similarly, the number of key events showed an increase from a baseline rating of 5.5 (SD 1.35) to 9.5 (SD 0.528), and the number of photos collected rose from an initial 5.2 (SD 1.39) to 9.7 (SD 0.48). Finally, the number of notes made on the photos increased from 5.2 (SD 0.92) to 9.7 (SD 0.48). These results demonstrate a significant improvement in all measured aspects of digital storytelling activities, reflecting enhanced participant engagement, storytelling complexity, and interaction with digital content over the course of the study.

Table 4 presents the mean attainment and changes in story sharing duration, number of key events, number of photos collected, and notes on the photos at baseline and the 2- and 4-week follow-ups among 50% (10/20) of the participants in the intervention group. The table illustrates a clear improvement in participants’ performance from the initial assessment to subsequent follow-ups. Substantial improvements were observed across all digital storytelling performance metrics, including longer storytelling durations, greater number of key events, more photos collected, and more notes recorded.

**Table 4:** Goal attainment ratings at baseline and the 2- and 4-week follow-ups and statistical comparisons.

Measure	Participant rating (1-10), mean (SD)				Researcher rating (1-10), mean (SD)
	Baseline (T0)	2 wk	4 wk (T1)		
Duration of story sharing	4.8 (1.23)	8.7 (1.34)	9.2 (0.79)	6.8 (1.93)	
Number of key events in the story	5.5 (1.35)	8.4 (1.43)	9.5 (0.53)	5.7 (1.06)	
Number of photos collected	5.2 (1.39)	8.6 (1.26)	9.7 (0.48)	7.3 (1.49)	
Number of notes on the photos	5.2 (0.92)	8.3 (1.77)	9.7 (0.48)	7.3 (1.49)	

In addition to the quantitative findings, qualitative observations during the field-testing phase highlighted noticeable changes in participants’ engagement and interaction patterns. Initially, several participants appeared hesitant to share their stories; however, by the third and fourth sessions, many became more active, showing greater willingness to discuss personal memories and respond to others’ narratives. Emotional expressions such as laughter, nostalgia, and mutual encouragement were frequently observed during group discussions. For example, one participant shared the following—“We chatted away, made new friends. Without this activity, I wouldn’t have met everyone, you know” (group 1; P1)—reflecting the social bonds that developed through the sessions. Another participant remarked the following—“I am very interested in this activity as it is a completely new experience for me. I have never done anything like it before” (group 2; P1)—highlighting the novelty and engagement stimulated by the Huiyou app.

Most participants also demonstrated increasing independence in navigating Huiyou, requiring less assistance over time. One participant expressed the following:

I managed to do everything by myself and didn’t ask for help from anyone.

Group 1; P2

Furthermore, the intervention appeared to strengthen interpersonal relationships beyond the sessions, with a participant noting the following:

I’ve had more interactions with my husband now; we reminisce about the old times together.

Group 1; P3

These observations provide supplementary evidence of improved social connectedness, storytelling engagement, and digital competency among participants with MCI.

No subgroup or sensitivity analyses were prespecified or conducted in this study. Given the small sample size and the exploratory nature of the trial, the analysis was limited to evaluating primary and secondary outcomes at the group level.

No harms or unintended events were reported in either the intervention or waitlist control group throughout the study period. Participants did not report any emotional distress, discomfort, or adverse reactions related to the digital storytelling sessions or study procedures. Facilitators also did not observe any concerning behaviors or incidents during intervention delivery.

## Discussion

### Principal Findings

The principal findings of this pilot randomized controlled trial investigating the effects of the mobile storytelling app Huiyou on social participation and storytelling ability among people with MCI are compelling. This study demonstrated that the experimental group, which engaged with the Huiyou app, exhibited a significant improvement in outdoor activities compared to the control group. This was reflected by a Cohen *d* value of 1.046, indicating a large effect size, suggesting that the intervention significantly increased the frequency and possibly the quality of outdoor social interactions among participants. In addition, there was a significant enhancement in interpersonal adaptation experiences, with a Cohen *d* of 1.290, also indicating a substantial effect. However, although there were strong effects observed for the LIFE-H total score, SCS-R total score, SWBS total score, interpersonal relationships, psychological balance experience, physical health experience, and target value experience, these improvements did not reach statistical significance. This may be due to the small sample size.

Despite the lack of statistically significant changes across all measures, the large effect sizes observed suggest meaningful improvements that warrant further investigation. These findings underscore the potential value of the Huiyou app in enhancing social participation among individuals with MCI but also suggest that larger-scale studies are necessary to fully assess the app’s impact. The intervention effectively increased the duration of story sharing, the number of key events detailed in the stories, the volume of photos collected, and the number of notes made on these photos. These improvements indicate an increase not only in participant engagement but also in the complexity and richness of the narratives shared by the participants. This multifaceted enhancement highlights the efficacy of the intervention in enhancing participants’ storytelling skills and their overall experience with the digital platform. It reflects a broader impact on their engagement and the quality of their interactions. This improvement suggests that the intervention successfully boosted participants’ ability to adapt to social interactions, enhancing their social integration and reducing feelings of isolation. These outcomes underline the potential of tailored digital storytelling interventions to enrich social engagement and overall quality of life for people with MCI.

### Comparison With Prior Work

#### Social Participation and Storytelling Engagement

In our discussion regarding the Huiyou intervention, we noted a significant increase in outdoor activities after the intervention, echoing the findings of Ryu and Heo [59]. They noted a positive correlation between life satisfaction and physical activities, suggesting the various benefits of outdoor engagement. The significant improvement in outdoor activities observed in the experimental group of this study compared to the control group highlights that the intervention led to increased participation in these activities by older individuals. The strong effect size (Cohen *d*=1.046) indicates a substantial impact of the intervention, demonstrating its effectiveness in enhancing outdoor activity among participants. This notable increase not only supports the efficacy of our intervention but also suggests that engaging in outdoor activities could be particularly beneficial for improving the overall well-being and social engagement of people with MCI. This finding is crucial for developing future interventions aimed at increasing physical activity and social interactions in this population. Our structured weekly meet-ups as part of the intervention encouraged active participation in the external environment, as highlighted in the study by Ryu and Heo [59]. Their research expands on this connection by linking outdoor engagement not only to physical and psychological well-being but also to increased life satisfaction and health perception. This indicates that the intervention’s impact on outdoor activities extends beyond immediate physical benefits, potentially influencing overall life satisfaction and health perception among participants, thus underlining the vital role of structured outdoor activities in improving quality of life for people with MCI. Furthermore, the holistic advantages of outdoor activities, bolstered by structured interventions, encompass physical health, optimism, and cultural engagement. This comprehensive approach to wellness emphasizes the importance of environmental factors in promoting outdoor activities, alongside the findings of Ryu and Heo [59] and Chang [60]. These studies highlight the multifaceted value of outdoor engagement in promoting health and well-being among people with MCI and stress the importance of both structured interventions and supportive environments in enhancing outdoor activity participation. The prototype developed in this study played a significant role in enhancing outdoor activities, particularly reflected in the notable differences in LIFE-H outdoor activity scores after the intervention. This aligns with existing research indicating the significant benefits of structured outdoor interventions, particularly those conducted in natural settings, for enhancing the physical and mental health of older adults. These studies highlight that such interventions can lead to improved physical activity, reduced stress, and better overall well-being [61]. These findings suggest that similar programs focusing on outdoor activities could be valuable in various contexts to promote a healthier lifestyle and greater social engagement. Specifically, rehabilitation programs that incorporate outdoor mobility have shown success in increasing activity levels, outdoor mobility, and endurance for older adults recovering from illness or injury. This suggests that incorporating structured outdoor activities into rehabilitation or wellness programs can have a notable impact on improving both physical endurance and quality of life for people with MCI [62]. Coming out of the room is a preliminary step toward engaging in outdoor activities. It is a crucial aspect of encouraging physical activity and social interaction, especially for people with MCI. By fostering a motivation to leave the room, interventions can lay the groundwork for enhanced outdoor activity, leading to improved health outcomes and overall well-being. Given this understanding, it is important to design interventions that address the barriers that may prevent individuals from coming out of their rooms. This might involve creating structured programs that are appealing and easily accessible, offering social incentives, and providing support to overcome potential anxiety or hesitation. Such efforts can increase the likelihood of engaging in outdoor activities, which in turn can lead to better physical health and more social connection.

Our findings on the Huiyou app underscore its substantial positive effects on interpersonal adaptation among participants with MCI. The significant improvement in interpersonal adaptation experience, highlighted by activities such as generating and sharing material, aligns with existing research on psychological interventions for MCI or dementia [63]. The intervention notably increased participants’ scores compared to those in the control group, which suggests that the digital storytelling approach not only enhances interpersonal skills but also supports people with MCI in adapting more effectively to social interactions. The strong effect size (Cohen *d*=1.290) emphasizes the impact of the intervention, illustrating its potential to significantly improve social adaptability in individuals with MCI. These findings advocate for the integration of storytelling elements into interventions as a powerful mechanism to enhance social capabilities and address some cognitive impairments associated with social challenges. Interventions that focus on building social connections and promoting social participation have been shown to improve interpersonal adaptation and reduce feelings of isolation [64]. Technology-based interventions such as digital applications that support reminiscence activities can further enhance memory recall and strengthen social interactions [20]. This aligns with our findings that the “generate material” phase, involving the searching and sharing of photos, facilitated increased interactions with friends and family, thereby improving social bonds.

Furthermore, Bentley et al [37] highlighted the importance of connecting with places to strengthen social contact, promoting a unified system with friends and family to share places and stories. This concept dovetails with our findings, wherein participants engaged in deeper reminiscence with their spouses to recall more detailed memories. This process not only nurtured more positive interactions but also effectively reduced negative exchanges, highlighting the value of digital storytelling interventions in cultivating meaningful interpersonal relationships. The convergence of our results illustrates the multifaceted impact of digital storytelling interventions on enhancing social interaction and communication. Through facilitating the creation and sharing of content, such interventions promote active social participation and reminiscence, contributing to improved interpersonal relationships among participants and their social circles.

Moreover, the study’s intricate assessment of digital storytelling activities revealed considerable improvements in engagement, complexity of storytelling, and interaction with digital content, marking a significant stride in enhancing participants’ quality of life. Digital storytelling interventions offer several benefits for older adults, including promoting mental health, building meaningful community connections, achieving digital literacy, mitigating negative ageism, and enhancing intellectual ability [65]. In addition, we found that these interventions can support self-expression and language expression, providing a platform for older adults to share their stories, which encourages active communication and engagement with others. By engaging in digital storytelling interventions, older adults can develop new skills, build social networks, and gain a sense of accomplishment, contributing to improved overall well-being. Given these observations, incorporating digital storytelling into rehabilitation and engagement programs could effectively improve participants’ quality of life through enhanced interaction and complexity in storytelling while also strengthening social engagement and psychological well-being. The Huiyou app notably enhanced participants’ storytelling engagement, as evidenced by increased narrative complexity and more active interaction with digital content. The structured sessions and targeted prompts encouraged deeper reflection and participation in storytelling activities. Furthermore, the process of generating and sharing stories fostered richer social interactions within participants’ networks, highlighting a positive potential toward improved social connectedness. Complementary structured outdoor activities also contributed to significant improvements in outdoor activity engagement, supporting both physical and psychological well-being. Overall, the findings suggest that structured interventions integrating digital storytelling, social participation, and outdoor activities can meaningfully enhance quality of life and social well-being for people with MCI. Although a detailed qualitative analysis is reported separately, key observations during the intervention reinforce the quantitative results. The progressive increase in storytelling engagement, emotional sharing, and group interaction suggests that Huiyou successfully fostered a supportive social environment for people with MCI. These behavior changes align with previous research emphasizing the role of peer interaction and shared memory activities in promoting social participation. Furthermore, the gradual improvement in participants’ digital literacy and confidence with the app underscores the potential for appropriately designed technology to support autonomy and reduce technology-related anxiety among older adults with cognitive impairment.

#### Self-Efficacy and Measurement Challenges

Our study examined the lack of significant changes in self-efficacy among older adults engaging in physical and social activities. It shed light on several critical factors that merit consideration. First, the intervention’s duration was a potential factor influencing these outcomes. Existing literature supports the notion that short-term interventions may not be sufficiently impactful to alter self-efficacy levels significantly, particularly in older adults who may have long-held beliefs about their capabilities [66]. Self-efficacy develops and is influenced over time through mastery experiences, vicarious experiences, verbal persuasion, and interpretations of physiological and emotional states [67]. Consequently, the relatively brief span of our intervention might not have provided ample opportunities for these processes to effectuate a meaningful change in self-efficacy levels. Second, the challenge of detecting significant changes in self-efficacy is compounded by the high baseline self-efficacy scores observed among participants and the possible insensitivity of the measurement tools used. This observation aligns with findings of other studies that highlight the difficulty in measuring subtle shifts in self-efficacy among populations with already high levels of this construct [66,68]. The necessity for more refined measurement tools is emphasized, suggesting a need for instruments that can discern minor yet impactful changes in self-efficacy. Such tools would enable a more accurate assessment of intervention effects, particularly in contexts in which initial self-efficacy levels are high. Third, the stress associated with participation in activities represents another critical consideration. Our findings indicate that stress may undermine self-efficacy beliefs, resonating with previous research indicating that stress and anxiety can negatively impact one’s confidence in performing future activities [69,70]. This means that incorporating strategies within interventions is crucial to managing stress and enhancing psychological resilience among participants. Thus, tailoring interventions to account for initial psychological states and using stress management techniques could be pivotal in supporting and possibly improving self-efficacy beliefs.

Given the cognitive and linguistic difficulties among people with MCI [71-73], measurement tools such as the SCS-R should be simplified for accurate evaluation. The linguistic complexity of some measurement tools, particularly the double-negative phrasing found in items of the SCS-R, may have posed cognitive challenges for participants with MCI. While this limitation was acknowledged, future research should pilot simplified or adapted versions of such instruments that are validated for populations with cognitive impairment to ensure accuracy and comprehension. Given these considerations, there is a pressing need for more straightforward or positively framed items in future studies, especially when assessing populations with cognitive impairments such as MCI. Simplifying the language used in measurement tools not only accommodates the cognitive profiles of these populations but also enhances the reliability and accuracy of the data collected.

While not all measures showed significant changes, certain areas such as physical health and self-acceptance exhibited positive trends toward improvement. This observation suggests that prototype-based interventions could contribute to a broader impact on these outcomes. Studies have shown that outdoor physical activity interventions in green and blue spaces can improve health outcomes, including well-being, mood, and physical performance [74]. Although our study indicated that structured activities play a pivotal role in enhancing outdoor activity engagement, the scope of these findings suggests the need for more extensive studies to validate these trends and determine the potential for these interventions to improve health outcomes across a wider range of indicators [75].

The results from the digital storytelling intervention suggest that further exploration in this field could lead to improved strategies for enhancing social participation and quality of life for people with MCI. The success of these interventions underscores the need to develop innovative approaches that effectively address the unique challenges faced by people with MCI.

### Limitations

Primarily, the pilot nature of this study, with 10 participants in both the intervention and control groups, serves as a foundational step, limiting the generalizability of our findings. The modest sample size also restricts our ability to detect significant differences across some outcome measures, underscoring the need for expanded research to validate these initial results in a future study. This study was conducted as a pilot trial to explore the feasibility and preliminary effects of a digital storytelling intervention for people with MCI. Although the study received ethical approval from the relevant institutional review board, it was not prospectively registered. At the time of initiation, trial registration was not required for minimal-risk behavioral interventions under institutional policy. We acknowledge the absence of prospective registration as a limitation and recommend that future confirmatory trials undergo prospective trial registration in accordance with best practices. Given the pilot nature of this study (N=20), the statistical power was limited, and the findings should be interpreted with caution. However, the promising trends observed in this preliminary investigation justify the design and implementation of a larger, fully powered randomized controlled trial to validate the intervention’s effectiveness and enhance generalizability to a broader population of people with MCI. Although randomization was used, a notable baseline difference in self-acceptance scores (SWBS) was observed between groups. This may have introduced bias into postintervention comparisons related to this variable. Future studies should consider stratified randomization or statistical adjustment to better control for such baseline differences. Concerning the sex distribution, our study observed a lower participation rate among men. Rather than reflecting a bias in recruitment or willingness to participate, this trend aligns with broader observations that, in China, men tend to engage less in collective activities compared to women. This aspect provides a cultural context to our findings and suggests avenues for further exploration into sex dynamics in social participation. A crucial point of consideration is the nature of the participants recruited through institutions, who tended to be more active or had previously engaged with psychological services. This selection bias suggests that the intervention’s preliminary findings may not fully represent the broader population of older individuals with MCI, particularly those who are less active. Recognizing this, there is a pronounced need for future studies to delve into the intervention’s effects on a more diverse and possibly less active older adult cohort, ensuring a comprehensive understanding of the intervention’s benefits across varying levels of initial activity and engagement. Addressing feasibility concerns, the potential commuting barriers highlighted the importance of local community engagement in facilitating participation. Organizing activities within participants’ communities could enhance the intervention’s accessibility and reduce logistical barriers to participation. We could also use an online platform for digital storytelling to support participants who live far away from the group members.

Moreover, the scalability of the Huiyou intervention requires careful consideration. Technical barriers such as digital literacy gaps among older adults, limited access to compatible devices, and challenges in maintaining user engagement over time could hinder broader implementation. Contextual challenges, particularly in low- and middle-income settings, include resource constraints and insufficient community infrastructure to support ongoing group activities. These considerations are crucial for ensuring that digital storytelling interventions such as Huiyou can be effectively scaled to benefit wider populations of people with MCI.

### Conclusions

The Huiyou intervention has shown promising effects on the well-being of people with MCI, significantly enhancing outdoor activities and interpersonal adaptation experiences. Notable improvements were observed in participants’ engagement with digital storytelling, contributing to more complex narrative constructions. Consistent with existing research, these outdoor activities were found to contribute to both physical and psychological health, potentially affecting overall life satisfaction. Enhanced social connections were facilitated through digital content sharing, boosting interpersonal relationships. However, improvements in self-efficacy were limited, suggesting challenges in enhancing this area through short-term interventions, particularly given the cognitive limitations associated with MCI. These findings underline the importance of developing suitable measurement tools that can capture nuanced changes in this population. 

Although most outcome measures did not reach statistical significance, the large effect sizes observed in outdoor activity and interpersonal adaptation outcomes suggest promising intervention potential. Given the limited sample size, these findings should be interpreted as preliminary, highlighting the need for further research using larger randomized controlled trials to substantiate and extend these early results. This study underscores the potential for digital interventions to improve quality of life for people with MCI and suggests a need for accessible and user-friendly digital platforms tailored to their specific needs. Future studies should expand on these findings by examining the intervention’s effectiveness across a more diverse and larger population to fully leverage digital tools in supporting individuals with MCI.

## Data Availability

Due to ethical considerations and data protection policies regarding vulnerable populations, including older adults with mild cognitive impairment (MCI), the deidentified participant data, data dictionary, and statistical code are not available for external sharing. Access is strictly limited to the research team as approved by the institutional ethics committee, and will not be granted to other researchers or institutions.

## Appendix

Multimedia Appendix 1 CONSORT checklist.

Multimedia Appendix 2 Independent-sample t test for change rates in key variables.

Multimedia Appendix 3 Baseline comparison of social participation.

## Abbreviations

GSES: General Self-Efficacy Scale
LIFE-H: Assessment of Life Habits
MCI: mild cognitive impairment
MoCA-C: Montreal Cognitive Assessment–Chinese version
SCS-R: Social Connectedness Scale–Revised
SWBS: Subjective Well-Being Scale
