# Supplementary material for: Effects of a Mobile Storytelling App (Huiyou) on Social Participation Among People With Mild Cognitive Impairment: Pilot Randomized Controlled Trial
Source: JMIR Hum Factors. 2025 Jun 18;12:e70177. doi: 10.2196/70177 (PMC12223459; doi:10.2196/70177)
Supplement: Multimedia Appendix 2 [file humanfactors_v12i1e70177_app2.docx]

Table 1 Independent sample t-test for change rates in key variables.

| **Variable** | ***t*** | ***df*** | ***p*** | **Cohen's *d*** |
| --- | --- | --- | --- | --- |
| LIFE-H Change Rate | 1.566 | 18 | 0.135 | 0.701 |
| SCS Change Rate | 1.562 | 18 | 0.136 | 0.698 |
| SWBS Change Rate | 1.16 | 18 | 0.261 | 0.519 |
| GSES Change Rate | -0.548 | 18 | 0.59 | -0.245 |
| LIFE-H Indoor activity Change Rate | 0.87 | 18 | 0.396 | 0.389 |
| LIFE-H Outdoor activity Change Rate | 2.339 | 18 | 0.031 | 1.046 |
| LIFE-H Interpersonal relationship Change Rate | 1.864 | 18 | 0.079 | 0.834 |
| LIFE-H Community life Change Rate | 0.242 | 18 | 0.811 | 0.108 |
| GSES Action Self efficacy Change Rate | -0.027 | 18 | 0.979 | -0.012 |
| GSES Coping Self efficacy Change Rate | -0.882 | 18 | 0.39 | -0.394 |
| SWBS Interpersonal adaptation experience Change Rate | 2.884 | 18 | 0.01 | 1.29 |
| SWBS Mental health experience Change Rate | 0.83 | 18 | 0.418 | 0.371 |
| SWBS Family atmosphere experience Change Rate | 0.845 | 18 | 0.409 | 0.378 |
| SWBS Psychological balance experience Change Rate | 1.259 | 18 | 0.224 | 0.563 |
| SWBS Physical health experience Change Rate | -1.307 | 18 | 0.208 | -0.585 |
| SWBS Target value experience Change Rate | 1.317 | 18 | 0.205 | 0.589 |
| SWBS Social confidence experience Change Rate | 0.762 | 18 | 0.456 | 0.341 |
| SWBS Contented and abundant experience Change Rate | -0.391 | 18 | 0.7 | -0.175 |
| SWBS Self-acceptance experience Change Rate | -0.283 | 18 | 0.78 | -0.127 |
