# Supplementary material for: Effects of a Mobile Storytelling App (Huiyou) on Social Participation Among People With Mild Cognitive Impairment: Pilot Randomized Controlled Trial
Source: JMIR Hum Factors. 2025 Jun 18;12:e70177. doi: 10.2196/70177 (PMC12223459; doi:10.2196/70177)
Supplement: Multimedia Appendix 3 [file humanfactors_v12i1e70177_app3.docx]

**Table 2**. Participants mean attainment, participants and changes in attainment at baseline, 2-week and 4‐week follow‐up.

| **Number of participants** | **Initial score** | **Follow-up 1** | **Follow-up 2** | **Change scores of follow-up 1** | **Change scores of follow-up 2** |
| --- | --- | --- | --- | --- | --- |
| 1 | 5 | 9 | 9.5 | 4 | 4.5 |
| 2 | 5.25 | 8.5 | 9.5 | 3.25 | 4.25 |
| 3 | 4.75 | 8 | 9.5 | 3.25 | 4.75 |
| 4 | 5.75 | 9 | 10 | 3.25 | 4.25 |
| 5 | 5.5 | 10 | 10 | 4.5 | 4.5 |
| 6 | 5.25 | 10 | 10 | 4.75 | 4.75 |
| 7 | 4.5 | 9.75 | 10 | 5.25 | 5.5 |
| 8 | 5 | 7.75 | 9 | 2.75 | 4 |
| 9 | 5 | 7.25 | 9 | 2.25 | 4 |
| 10 | 5.75 | 5.75 | 8.75 | 0 | 3 |
| Average | 5.175 | 8.5 | 9.525 | 3.325 | 4.35 |
